# Procrustes-based geometric morphometrics on MRI images: An example of inter-operator bias in 3D landmarks and its impact on big datasets

**DOI:** 10.1371/journal.pone.0197675

**Published:** 2018-05-22

**Authors:** Amro Daboul, Tatyana Ivanovska, Robin Bülow, Reiner Biffar, Andrea Cardini

**Affiliations:** 1 Department of Prosthodontics, Gerodontology and Biomaterials, University Medicine Greifswald, Greifswald, Germany; 2 Institute of Biophysics, Georg-August-University, Göttingen, Germany; 3 Institute for Radiology and Neuroradiology, University Medicine Greifswald, Greifswald, Germany; 4 Dipartimento di Scienze Chimiche e Geologiche, Università di Modena e Reggio Emilia, Modena—Italy; 5 School of Anatomy, Physiology and Human Biology, The University of Western Australia, Crawley WA, Australia; Università di Roma, ITALY

## Abstract

Using 3D anatomical landmarks from adult human head MRIs, we assessed the magnitude of inter-operator differences in Procrustes-based geometric morphometric analyses. An in depth analysis of both absolute and relative error was performed in a subsample of individuals with replicated digitization by three different operators. The effect of inter-operator differences was also explored in a large sample of more than 900 individuals. Although absolute error was not unusual for MRI measurements, including bone landmarks, shape was particularly affected by differences among operators, with up to more than 30% of sample variation accounted for by this type of error. The magnitude of the bias was such that it dominated the main pattern of bone and total (all landmarks included) shape variation, largely surpassing the effect of sex differences between hundreds of men and women. In contrast, however, we found higher reproducibility in soft-tissue nasal landmarks, despite relatively larger errors in estimates of nasal size. Our study exemplifies the assessment of measurement error using geometric morphometrics on landmarks from MRIs and stresses the importance of relating it to total sample variance within the specific methodological framework being used. In summary, precise landmarks may not necessarily imply negligible errors, especially in shape data; indeed, size and shape may be differentially impacted by measurement error and different types of landmarks may have relatively larger or smaller errors. Importantly, and consistently with other recent studies using geometric morphometrics on digital images (which, however, were not specific to MRI data), this study showed that inter-operator biases can be a major source of error in the analysis of large samples, as those that are becoming increasingly common in the 'era of big data'.

## Introduction

Procrustes-based geometric morphometrics (PGMM) is the leading methodology in modern morphometrics [1–3]. Its flexibility and relative simplicity make this powerful method suitable for applications to a variety of images, such as the many types of digital images, which have become increasingly common in medicine and other fields of science. Among these, magnetic resonance imaging (MRI) is one of the most interesting examples of computerized anatomical images, as it allows to see both soft and hard-tissues and to perform quantitative investigations in either 2D or 3D. Although computed tomography (CT) might offer higher resolution and accuracy of hard tissues, it is considered as an increasing source of radiation and its use for research purposes is prohibited in most countries and is associated with many ethical issues due to radiation exposure and the subsequent effects it carries on humans. The use of MRI eliminates the exposure to the ionizing radiation encountered in CT and it is considered as a safe and robust imaging modality that can be incorporated into epidemiological and population-based studies.

The potential of MRI data has been promptly recognized in modern morphometrics, that employed it for some of the first pioneering biomedical applications of PGMM. As follows, Bookstein and colleagues performed a series of analyses on the shape of the corpus callosum using sagittal brain MRIs [4–6]. They were able to find characteristic traits associated with developmental and neurological anomalies, including increased variance in individuals affected by schizophrenia and a morphological ‘signature’ typical of the fetal alcohol syndrome. This work helped to move beyond simple measurements of size (inter-landmark distances, volumes etc.) in diagnostic MRI studies and to explore shape variation in relation to disease and other factors. In fact, although landmark-based approaches to the analysis of MRI data has been mainly performed, until now, in a biomedical context, as technology improves and instruments become more accessible, MRIs could provide useful data in many other disciplines such as, among others, evolutionary biology[7] and the study and conservation of museum specimens[8].

In all quantitative investigations, measurement error is a crucial aspect to be investigated and carefully considered in designing a study. This is becoming even more central in the era of ‘big data’ and digital data sharing, as large samples from longitudinal population studies, and big datasets obtained by merging information from multiple centers, become available, offering new exciting avenues for powerful analyses. However, at the same time, 'big data' often imply a degree of heterogeneity in how data are acquired. This happens not only when different datasets are merged, but also in long-term investigations, such as in longitudinal studies, in which different instruments and/or operators are used to collect the data over time.

In general, and especially in recent years, there has been a renewed interest in studies of measurement error using images for PGMM analyses. Arnqvist & Mårtensson[9] and Klingenberg et al.[10] have been among the first studies to clearly frame protocols for the assessment of measurement error in PGMM. Viscosi & Cardini[11] exemplified some of their suggestions in the context of taxonomic studies on plant leaves. Fruciano[12] provided an extensive review on a variety of methods for the analysis of measurement error using Procrustes shape data, while von Cramon-Taubadel[13] mostly focused on landmark precision. Even more recently, Fruciano et al. [14] and Shearer et al.[15] emphasized the importance of errors arising from combining PGMM data from multiple operators and different devices. Both studies suggested that inter-operator errors could be particularly important, often larger than intra-operator variability and typically much larger than differences related to the type of device used to acquire the data. They also suggested that increasing operator expertise and/or focusing on subsets of precise landmarks might help to reduce the effect of measurement error. Nevertheless, Shearer et al.[15] showed, using specimens of multiple species of macaques, representing well separated and fairly distant evolutionary lineages, that variation due to inter-operator differences could be substantial. In this respect, Fruciano et al. [14] reported comparatively smaller inter-operator variability (ca. 8–12% of the total sum of squares for shape) but focused on a higher taxonomic level (macropodoid marsupials including eight different genera), and thus likely had a much larger total variance in their dataset.

Measurement error is routinely quantified in morphometric analyses using MRI data[16–18]. However, this assessment is rarely done within the framework of PGMM and no study using landmarks obtained from MRI seems to have focused on inter-operator differences in estimates of Procrustes size and shape in relation to sample variance. As measurements are made often by different operators, the correspondence between landmark data digitized by different operators on the same sample of individuals is termed as “reproducibility” [19, 20]. As with any type of measurement error, reproducibility can be assessed in absolute or relative terms. In the first case, one estimates the absolute differences of the replica measurements; in the second instance, however, differences are related to the 'true' variability among subjects in the sample. Sometimes, this second type of assessment is referred to as “reliability”[19].

In the present study, we used PGMM on a configuration of soft and hard tissue 3D facial landmarks that were digitized by different operators on MRI images from a sample of adult individuals of Caucasian ancestry. We assessed the amount of digitizing error among the operators using either the full set of landmarks or the two subsets of respectively hard- and soft-tissue landmarks. Throughout the study, our main focus was on the precision of measurements, which refers to “the extent to which repeated observations conform”[20]. Therefore we only assessed the component of measurement error related to differences in landmark data digitized by different operators on the same MRI images. As mentioned earlier, this potential bias is often the largest component of measurement error in PGMM analyses[14, 15] and will likely become even more crucial in the context of analyses of big datasets, where measurements are rarely collected by a single individual. Therefore, besides exemplifying the assessment of inter-operator differences using multiple digitizations of landmarks on the same sample of MRI data, we will explore how using multiple operators might affect the results of analyses in a population based cohort of more than 900 individuals, and to what extent does the inter-operator error influence the analysis in large population based MRI studies.

## Materials and methods

### MRI data acquisition and image analysis

The head scans were performed on a 1.5-T magnetic resonance system (Magnetom Avanto; Siemens Medical Solutions, Erlangen, Germany) as a part of a whole-body MRI scan protocol. The complete protocol was previously described elsewhere[21, 22].

The ethics committee of University of Greifswald approved the study protocol, and written informed consents were obtained from all the subjects who agreed to participate.

The analysed head scans comprised of an axial T1-weighted head scans (ultra-fast gradient echo sequence using the following imaging parameters: repetition time of 1900 ms; echo time of 3.37 ms; flip angle of 15^o^; matrix of 176 × 256 × 176 and a voxel size of 1 × 1 × 1 mm. The post-processing of the axial T1-weighted sequences comprised multi-planar reconstruction (MPR) with 1 mm slice thickness for further image interpretation and was performed automatically by the viewing software Osirix (Osirix version 3.8.1. Pixmeo, Geneva, Switzerland).

**Fig 1:**
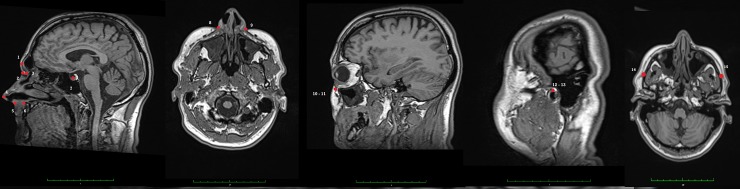
Landmark detection using multi planar reconstruction (MPR) with axial view as the centre of orientation. Plotted landmarks: 1. Glabella, 2. Soft Nasion, 3. Hard Nasion, 4. Pronasale 5. Subnasale 6. Anterior nasal spine, 7. Sella 8 & 9. Alare 10 & 11. Orbitale 12 & 13 Porion 14 & 15 Zygion.

Landmarks were digitized by three different operators on those images in Osirix. All operators were dentists with previous training on the use of Osirix software and craniofacial landmark identification. For investigator blinding, the images were identified by code and analyzed anonymously in random order. The landmark configuration (Fig 1) was selected to focus on adult facial variation in humans. Strictly speaking, Sella (landmark Nr. 5) is not part of the facial skeleton but was included as it helps to relate facial proportions to the anterior cranial base region. Each digitized landmark gave positional values in the form of (x,y,z), whereby the X-axis represented the right-left direction, the Y-axis and Z-axis corresponded to the posterior-anterior and superior-inferior directions respectively.

Inter-operator differences were assessed in 20 women randomly selected from a larger sample. The MRI sequences were assessed by three blinded operators, thus producing a sample of 60 observations (for brevity, called the “replica sample”). A single sex was chosen to focus on a highly homogeneous sample. Also, women tend to be smaller than men, thus making errors in size potentially more relevant [23].

A larger sample (“study sample”) was also used to explore whether possible differences among operators may produce an evident bias in a big dataset. This sample contained 906 adult individuals of both sexes digitized by the same three operators (each measuring different study subjects). More precisely, female/male sample sizes split by operators (OP1, OP2 and OP3) were: OP1) 102/61; OP2) 106/163; OP3) 252/222).

### Procrustes-based geometric morphometrics

Landmark data were analysed using geometric morphometrics[2, 3]. 3D anatomical landmarks were aligned using a Procrustes superimposition[24]. This mathematical procedure estimates size as the square root of the sum of squared distances from the centroid of a landmark configuration. This is called centroid size (CS), although we will often informally refer to it simply as “size”. Thus, size was computed for each individual in a sample, removed from the original data, and the resulting ‘size-free’ coordinates were superimposed by overlapping the centroids of all individuals and by minimizing the sum of inter-landmark squared distances in the sample. These operations took size out of the original raw coordinates and reduced irrelevant positional differences. The resulting new coordinates are called Procrustes shape coordinates. The magnitude of their differences in the multivariate shape space is measured by the Procrustes shape distance. In most biological applications this distance is a good approximation of the Riemannian Procrustes Distance computed on the curved manifold [1].

### Landmark reproducibility

Reproducibility was assessed in three steps: (I) the estimation of absolute error for the raw coordinates of each landmark, similarly to previous studies of MRI data[17, 18]; (II) the quantification of size and shape reliability and the proportion of inter-operator differences in the replica sample; (III) the exploration of the impact on the analysis of size and shape using data collected by different operators in the larger study sample of 906 men and women.

In the first step (analysis I) we preliminarily explored whether there are differences depending on the tissue type (soft or hard) on which landmarks are digitized. Analyses (II) and (III) were performed on the total configuration of 15 landmarks, as well as separately on the 10 hard-tissue facial landmarks and on the five nasal soft-tissue ones but focused on size and shape obtained using PGMM.

#### (I) Absolute error

Because landmarks were digitized by all operators on the same MRI sets, it was possible to quantify the absolute inter-operator digitizing error of each landmark in any individual. This was achieved by computing, for each landmark in each of the 20 individuals, the variation of the distances between each landmark and its average computed from the three replicas. For instance, for landmark 1 (L1) on individual 1, using the raw coordinates, we computed the distance in mm of L1 digitized by the first operator from the average position of L1 measured by all three operators, and then we did the same for the second and third operators, thus obtaining the operator deviations (ODEV) from the centroids. ODEV was used to assess if one or the other operator had larger absolute errors, as well as to summarize the average deviation (AVEDEV) of all three operators. To this aim, to obtain AVEDEV, we computed the mean of the three ODEV of a given landmark.

#### (II) Reproducibility of size and shape in the 20 women sample

The replica dataset was analysed as explained in Viscosi & Cardini[11] and Fruciano[12]. Thus, using a Procrustes ANOVA (analysis of variance), we partitioned variance in size or shape among individuals (i.e., averaged replicas representing the ‘true’ sample variance) and residual component (i.e., variation among replicas).

The ANOVA tests whether individual variation is significantly larger than error. However, it should be noted that with this type of test, highly significant individual variation can be found despite large measurement error (including significant biases: e.g. Fruciano et al [14]. Therefore, we mainly focused on estimates of effect size. To this aim, we used the proportion of sum of squares accounted for by each factor (individual and measurement error), which we will refer to as R2, and also used the mean sum of squares from the ANOVA to calculate the intraclass correlation coefficient, ICC[12]. ICC offers an alternative, but related method to R2, for assessing how much variation can be attributed to ‘true’ individual differences, with ICCs closer to 1 suggesting almost perfect reproducibility and thus smaller measurement error[19]. R2 and ICC are computed similarly for size and shape, but for shape the computations are fully multivariate and therefore simultaneously assess reproducibility across all landmarks and shape coordinates, as appropriate for Procrustes data[11, 12].

Reproducibility was also explored using correlational analyses and summary diagrams. Correlations provide an additional tool for exploring congruence among operators but cannot be used to investigate biases[20]. Thus, they should be interpreted with caution and only in relation to findings from other analyses such as R2s and ICCs from the ANOVAs. For size, we used pairwise (inter-operator) Pearson correlations together with the corresponding scatterplots of centroid size. For shape, we computed, pairwise between all operators, matrix correlations of Procrustes shape distances. Also, Procrustes shape similarity relationships in the replica sample were graphically summarized using a UPGMA (unweighted pair group method using arithmethic average) phenogram. In this tree diagram, replicas by different operators should cluster ‘within individual’ in triplets or at least in pairs, if inter-operator differences are small (i.e., high reproducibility and small measurement error).

Finally, R2 for the effect of operators (i.e., using operators as a grouping variable in an ANOVA) were used to approximately estimate the magnitude of potential inter-operator biases. This analysis was done for both size and multivariate shape.

#### (III) Inter-operator bias: Exploratory analysis in the 906 individuals sample

The ANOVA R2, that estimates the variation accounted for by operators as a grouping factor (as explained in the last paragraph of the previous section) was employed as an approximate quantification of the inter-operator bias in the total 906 individuals sample. As both men and women were present in the study sample, the hierarchical ANOVA was run adding sex as a main factor above the level of operator. This allowed to control for the effect of sex on total variance before estimating inter-operator differences. It also provided a ‘biologically meaningful’ factor against which to compare the magnitude of the inter-operator error. In the absence of inter-operator biases (and using fairly balanced samples—see Discussion), the expectation is that there should not be any appreciable inter-operator difference. In contrast, as men and women display sexual dimorphism in facial morphology[25] (e.g., Fink et al., 2005), sex is a genuine factor that, in a large sample, should explain a significant amount of variance. Thus, the operators' R2 should be clearly smaller than the R2 of sex.

Software

Analyses were mainly performed in R (2017) using the packages Vegan[26], Geomorph[27] and Morpho[28]. However, absolute errors (and the corresponding profile plots) were computed in a spreadsheet using Gnumeric (http://www.gnumeric.org/); the Procrustes ANOVA was performed both in R (2017) and MorphoJ 1.06d[29]; correlational analyses, scatterplots and phenograms were done in in PAST 2.17c[30].

## Results

### (I) Absolute error in the 20 women sample

**Fig 2:**
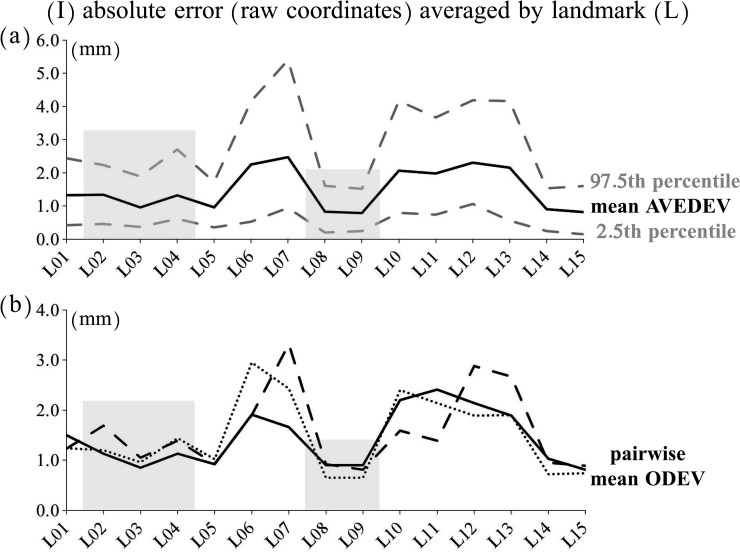
(I) Absolute error: AVEDEV and ODEV averaged for each landmark across all 20 individuals; soft-tissue landmarks are emphasized using a grey background: a) mean AVEDEV shown with a solid black line with its 2.5^th^-97.5^th^ percentiles shown using broken grey lines; b) mean ODEV: solid line, operator 1; broken line, operator 2; dotted line, operator 3.

AVEDEVs and ODEVs showed a similar trend of variation among operators and across landmarks (Fig 2), as expected form correlated measurements (r≥0.88). Both ranged on average from slightly less than 1 mm to ca. 3 mm, with the largest absolute deviation being almost 6 mm. However, some landmarks clearly had smaller error and higher reproducibility, and these included all the nasal soft-tissue landmarks plus a few bone landmarks. On average, absolute errors estimated using both metrics were approximately 65% larger in bone compared to soft-tissue landmarks.

### (II) Reproducibility of size and shape in the 20 women sample

Table 1 summarizes the results of the Procrustes ANOVA testing individual variation relative to measurement error (i.e., the differences between replica digitization done by the three operators). In all cases (size and shape, and different landmark configurations) individual differences accounted for significantly more variance than error. However, both R2 and ICC congruently showed that reproducibility varied widely depending on the type of data being analyzed.

**Table 1:** Reproducibility (analysis II) of size and shape in the 20 women sample with three replicas: Procrustes ANOVA comparing individual variation, in centroid size and shape, to measurement error.

data	landmarks	factor	SS	MS	df	F	P	R2	ICC	Pillai_tr.	P
CS	all	indiv.	2548.6	134.1	19	87.55	< .0001	98%	0.98	-	-
		error	61.3	1.5	40			2%			
		total	2609.9								
	bone	indiv.	1905.4	100.3	19	61.06	< .0001	97%	0.97	-	-
		error	65.7	1.6	40			3%			
		total	1971.1								
	nose	indiv.	458.2	24.1	19	9.68	< .0001	82%	0.81	-	-
		error	99.6	2.5	40			18%			
		total	557.8								
SHAPE	all	indiv.	0.1467	0.000203	722	5.46	< .0001	72%	0.69	17.46	< .0001
		error	0.0566	0.000037	1520			28%			
		total	0.2032								
	bone	indiv.	0.1314	0.000301	437	4.42	< .0001	68%	0.63	13.34	< .0001
		error	0.0626	0.000068	920			32%			
		total	0.1940								
	nose	indiv.	0.2995	0.001970	152	9.56	< .0001	82%	0.81	5.74	< .0001
		error	0.0660	0.000206	320			18%			
		total	0.3655								

In general, reproducibility was very high or fairly high for size. The full configuration and the bone landmarks were, in this respect, better than nasal soft-tissue landmarks with errors in size estimates accounting for 2–3% of variance compared to 18% for the nose. This trend was, however, reversed for shape data. With shape, reproducibility was low for the full and bone landmark configurations (with errors accounting for about 30% of total variance) and moderately high for nasal soft-tissue landmarks (less than 20% of variance due to measurement error).

**Fig 3:**
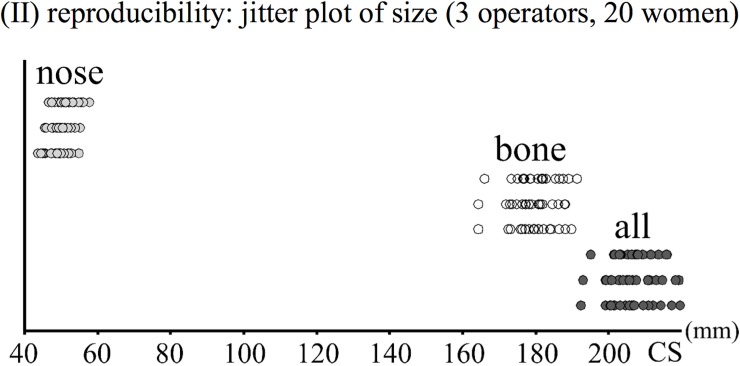
(II) Reproducibility of centroid size visualized using jitter plots for the three sets of landmarks (nose, bone and all landmarks) using estimates from the three operators.

**Fig 4:**
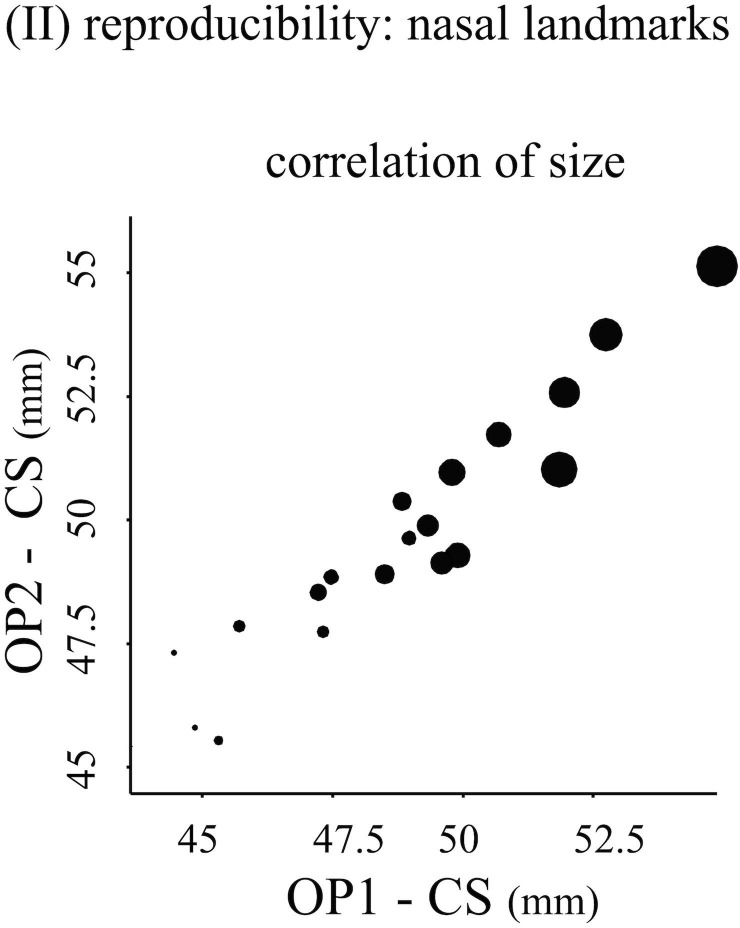
(II) Reproducibility of size: Scatterplot of nasal size used as an example of the graphical exploration of similarities across different operators: Operators 1 and 2 are shown respectively on the horizontal and vertical axes, while the size of the circles is proportional to size estimated from operator 3.

The generally high reproducibility of size was consistent with the correlational analyses (Table 2, Figs 3 and 4). Inter-operator pairwise correlations were always high (≥ 0.93) and only slightly smaller for the nose. In contrast, correlations of Procrustes shape distance matrices varied more across landmark configurations, with bone landmarks having the lowest inter-operator pairwise correlations (on average, r = 0.62), and the total and nose configurations having on average intermediate (r = 0.75) and highest (r = 0.81) correlations respectively.

**Table 2:** Reproducibility (analysis II) of size and shape in the replica sample: Between operators pairwise correlations of centroid size (Pearson correlation) and shape (correlation of shape Procrustes distance matrices).

	landmarks	all		bone		nose	
data	operators	OP2	OP3	OP2	OP3	OP2	OP3
CS	OP1	0.98	0.98	0.98	0.96	0.96	0.98
	OP2	-	0.99	-	0.98	-	0.93
	averaged	0.99		0.97		0.95	
SHAPE	OP1	0.77	0.73	0.65	0.65	0.81	0.82
	OP2	-	0.75	-	0.56	-	0.82
	averaged	0.75		0.62		0.82	

**Fig 5:**
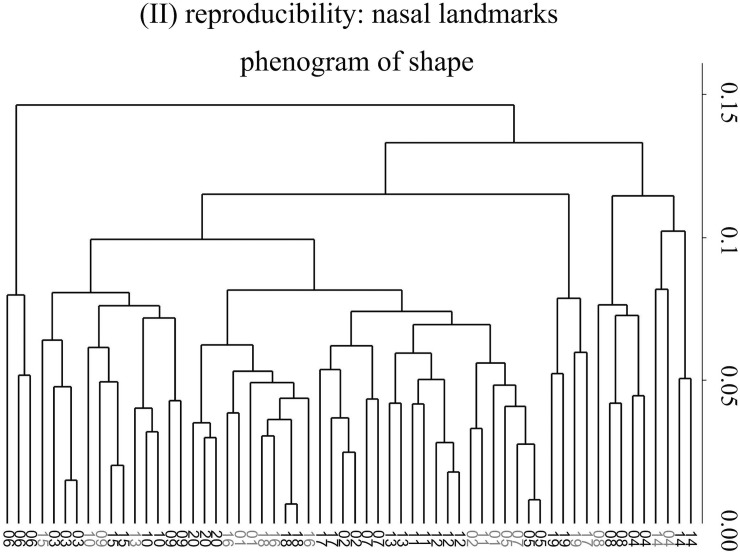
(II) Reproducibility of shape: UPGMA phenogram based on Procrustes shape distances for the 20 women (each indentified by a progressive number from 1 to 20) digitized by all three operators: With high reproducibility, all three replicas, or at least two of them, should cluster together 'within' individual (black numbers); when this does not happen, numbers are shown using light grey. As in Fig 4, nasal data are used as an example.

In the total shape phenogram (not shown), 12 out of 20 cases clustered in triplets of replicas, ‘within individual’, using all landmarks, but that happened only four times for both the bone (not shown) and nose (Fig 5) subsets of landmarks. However, for the nose and total configurations, at least two of the three replicas clustered as nearest neighbours in respectively 17–18 of the 20 individuals, while for the bone landmarks that happened only in 13 of the 20 women sample.

Finally, the variance accounted for by inter-operator differences (Table 3) in the replica sample was less than 2% for size in the total and bone configurations, but more than 13% in all other cases using either size (nose) or shape (all three configurations). Indeed, the nose stood out for the large inter-operator differences in size but also showed smaller variation among operators for shape (13% *vs* 15–18% in respectively the total and bone configurations).

**Table 3:** Inter-operator bias (analyses II-III): R2 of centroid size and shape estimated in ANOVAs using operator as a grouping factor in the replica sample and the study sample. For the study sample, R2 for sex, as main factor above operator, is also shown.

		replica sample*			study sample (N = 906)		
data	factor	all	bone	nose	all	bone	nose
CS	sex	-	-	-	57%	53%	40%
	operator	1%	1.5%	15%	0.1%	0.3%	7%
	residual	99%	99%	85%	43%	47%	53%
SHAPE	sex	-	-	-	2%	1%	5%
	operator	15%	18%	13%	6%	7%	2%
	residual	85%	82%	87%	92%	92%	93%

*For the replica sample, the R2 for individuals is not reported as it is in fact both individuals and replicas.

### (III) Inter-operator bias: Exploratory analysis in the 906 individuals sample

When we explored the magnitude of sex ('true' effect) and inter-operator (bias) differences in the study sample of 906 men and women (Table 3), we found that sex accounted for between 40% (nose) and almost 60% (all landmarks) of size variance, but only 1% (bone) to 5% (nose) of shape variance. In contrast, inter-operator differences were tiny for size, with the exception of the nose, where they accounted for 7% of sample variance (controlled for—i.e., unrelated to—sex). This means that the magnitude of sex differences in size was hundreds of times larger than inter-operator variation using all landmarks or just those on the bone. Even for the nose, despite fairly large differences among operators, sex accounted for six times more variance than operators. Thus, overall, inter-operator biases were mostly negligible for size compared to sexual dimorphism.

**Fig 6:**
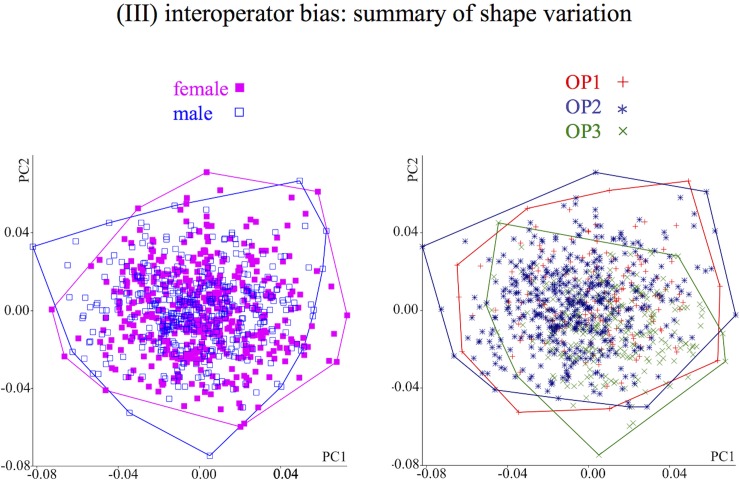
(III) Inter-operator bias: Scatterplots of the first two PCs (principal components) of total shape (all 15 landmarks) accounting for respectively 15.5% and 11.0% of total variance; sex (a) and operator (b) are shown using different symbols. Despite PCs being computed regardless of a priori groups, operators (a meaningless grouping factor in the absence of bias) show less overlap than sexes (i.e., biological groups).

Results were remarkably different for shape using all landmarks or just those on bones. Here, inter-operator differences accounted for 6–7% of total variance, which means that, contrary to size, operators' error were larger than sexual dimorphism, accounting for three to seven times more variation than sex. This was also suggested by Fig 6, where groups were plotted on PC1-PC2 of shape for the total configuration, as an example. This figure showed that, despite PCs maximizing total variance regardless of any a priori group, operators displayed a degree of separation whereas females and males overlapped almost completely. PC scatterplots (not shown) of bone shape produced the same pattern (not shown) as the total configuration, whereas those of the nose did the opposite, suggesting some separation between sexes and large overlap among operators. Indeed, for the nose, contrary to the other two configurations, results for shape were comparable to those of size, with sex accounting for three times more variance than operators, which only accounted for 2% of non-sex-related variance.

In summary, the outcome of the exploratory analysis of inter-operator bias in the study sample (III) was in good agreement with results from the reproducibility study (II): measurement error due to operator differences was negligible for size in all datasets, although more pronounced in the nose soft-tissue landmarks; however, despite significantly larger variation among individuals compared to differences among replicas (II), measurement error was large for shape with operator bias accounting for more variance than sex differences in two of the three configurations. The main exception for shape was the nose, that consistently with the relatively small absolute error (I) of its soft-tissue landmarks, showed approximately similar amounts of measurement error in both size and shape (II), with sex variance three to six times bigger than operator differences (III).

## Discussion

### Preliminary considerations

In this study, we assumed that instrumental errors, that we did not assess, were likely to be negligible compared to the inter-operator differences. Indeed, we found that inter-operator absolute errors in our study were more than one order of magnitude larger than scanners' errors generally reported in previous work. For instance, Figs 1–3 of Han et al.[31] show that absolute differences in cerebral cortical thickness related to different manufacturers or MRI field strength, as well as to differences in smoothing parameters, are likely to be in the order of a fraction of a mm (ca. 0.02 to 0.3 mm). In contrast, inter-operator absolute errors in our analyses ranged on average from about 1 to 3 mm. These estimates are consistent with those of Maudgil et al.[18] and Chollet et al. [17], who reported average absolute errors of 1–2 mm or more. Thus, as assumed, it does seem that MRI acquisition and processing introduce a source of measurement error which is negligible compared to inter-operator differences in landmark coordinates.

Implicitly, we also assumed that inter-operator differences indirectly address also the issue of intra-operator variability. This is plausible, as the 15 landmarks used in the study are a subset from a larger set of points from which low precision landmarks had been excluded[32, 33]. Also, it is reasonable because generally inter-operator differences are at least as large as the intra-operator variability plus, potentially, an extra amount of variation due to the fact that different observers might consistently place at least some landmarks in slightly different positions. This type of bias has been found for instance by Fruciano et al.[14] using PGMM analyses of 3D surface models of marsupial crania. Similarly, Shearer et al. [15] observed that “inter-observer error was consistently higher than all other potential error types observed among researchers” in their study of 3D landmarks from surface scans of macaques. Chollet et al.[17], however, surprisingly found smaller absolute differences between operators than among replicas by the same operator but, in agreement with our study, and consistently with the conclusions of Fruciano et al.[14] and Shearer et al.[15], Maudgil et al.[18] found inter-operator absolute errors on average about two times bigger than intra-operator ones.

Thus, even if it is always desirable to assess all components of measurement error, including differences in methods or devices for image acquisition and intra-operator variability[9], it seems likely that we quantified the largest source of error in our dataset and probably in similarly acquired sets of data.

Although inter-operator differences could have been explored in our main study sample of 906 subjects, no statistical test of the bias was possible. This type of test requires replicated readings and can be done pairwise between operators, using a paired t- test, or simultaneously for all three operators, using a repeated measures ANOVA (or for shape, the multivariate version of these tests should be used). In fact, we could have performed such tests in the replica sample, but we chose not to do it. This is because, as in the Procrustes ANOVA, we were more interested in the relative magnitude of measurement error and much less in whether that is statistically significant.

Another cause for caution before testing inter-operator differences in the main study sample was that the data was partly unbalanced. The three operators in this study digitized different numbers of individuals and only OP3, who digitized almost half of the data, measured similar numbers of men and women. In contrast, OP1 measured more females than males and OP2 did the opposite. For this reason, when operator differences in such heterogeneous samples are large (i.e., mainly in shape from the bone and total landmark configurations), estimates of sexual dimorphism could have been slightly inflated. On the other hand, for the same reason (i.e., unbalanced samples), one cannot exclude that the opposite happened, that is that inter-operator variability was slightly overestimated because one operator digitized more men and another more women. Indeed, this is more likely to have happened for size, where sexual dimorphism is much larger (R2 between 40% and almost 60%) than differences between operators.

Overall, however, the effect of unbalanced sampling in the exploratory analyses of the study sample should be small. This is because, as said, OP3 measured almost balanced samples, but also because the sex bias in the other two operators was small. Besides, in terms of estimates of inter-operator differences, results from the study sample (III) are supported by those from the replica sample (II), which was perfectly balanced and only included one sex: both analyses (Table 3) showed a relatively modest amount of error in nasal size and shape, a totally negligible error in size using either all or just the bone landmarks and a proportionally large amount of shape variation due to operator biases in the same two configurations. Finally, that estimates of sex and operator effects are likely to be accurate regardless of the unbalanced samples was suggested by repeating the ANOVAs (results not shown) after randomly selecting 50 individuals within each sex and operator (i.e., total N = 300) to have a fully balanced design: the resulting estimates of R2 for sex and operator from the perfectly balanced analyses were very similar to those from the unbalanced samples (r>0.99, with largest difference in R2 between balanced and unbalanced design being respectively for operator 8.7% *vs* 6.8% and for sex 31.7% *vs* 40.5%, both found in data from nasal size, and absolute differences across all datasets and effects being on average just 1.4%).

Finally, as a conclusion to the preliminary considerations, we would like to remark that, when analyses are performed on symmetric structures, it is possible to further split total variance into symmetric and asymmetric components using the Procrustes ANOVA[10]. We did not do it as we were not specifically interested in asymmetry in this study and chose to keep the design of the ANOVA simple. However, researchers interested in patterns of asymmetry could further partition variance but would have to assess (at least in a subsample with replicas) that asymmetry is larger than measurement error, which is especially important for fluctuating asymmetries (i.e., small non-directional differences between left and right sides). This is, for instance, exemplified by the recent paper of Fruciano et al.[14]. In their analysis on kangaroo crania, they found that fluctuating asymmetry was significantly larger than measurement error only using a subset of higher precision landmarks. However, it is interesting to observe that, even in that case and despite statistical significance, R2 for fluctuating asymmetry was about 1% whereas total error (device plus operator) accounted for more than 4% of total variation.

If in our replica sample we had done the same type of variance partitioning for symmetric and asymmetric components (results not shown), we would have been in a situation similar to Fruciano et al. [14] for the total and bone configurations, with fluctuating asymmetry significantly larger than error despite being about 1/3 of its magnitude when estimated using R2. For the nose, in contrast, fluctuating asymmetry would have been significant and of similar magnitude as inter-operator differences, both accounting for approximately 18% of variance. In cases when, despite significance, the size of the effects being tested is small or similar (in relative terms), the decision whether or not to interpret one or the other effect becomes more difficult and, if done, the problem should be explicitly stated and great caution in the interpretation should be suggested. Especially in small samples, the use of resampling methods based on tests that do not assume isotropic variation around landmarks[10] (Klingenberg et al., 2002) may lead to more reliable tests, whereas increasing the number of landmarks may not necessarily produce an improvement[34].

### Why different amount of error in size and shape across sets of landmarks?

In the previous subsection we clarified a few points and most importantly that inter-operator differences are likely to be the main source of measurement error and results seem fairly robust with replica and study samples providing largely congruent answers. Focusing now on the interpretations of these findings, one of the most evident outcomes of the study is that all analyses consistently indicated that soft-tissue nasal landmarks are less strongly affected by error (especially in absolute terms and in relation to shape variation). At a first glance, this may look counter-intuitive, as we might expect that landmarks on bones should be more precise and easy to locate than landmarks on soft-tissues. However, one has to consider the specific type of image data being used and, in fact, probably the simplest explanation of why soft-tissue landmarks are so reproducible is in relation to how MRI works. As stated by Morooka et al.[35]: “In biological tissues, MRI signals are generated by hydrogen atoms, with water and fat content accounting for the majority of the signal. All soft-tissues and cancellous bone contain a large fraction of water, so the magnetic susceptibility can be approximated by that of water. In contrast, cortical bone and air do not generate significant MRI signals. Nevertheless cortical bone can distort magnetic fields in nearby tissues that do generate MRI signals, thereby resulting in geometric distortion near these interfaces”. The operators in this study found that the soft-tissue landmarks on MRIs have better visibility and are easier to locate and therefore may produce smaller errors and higher reproducibility, something that had already been shown, for instance, on knee joint measurements by Wilcox et al.[16].

If soft-tissue landmarks on MRI are more precise in absolute terms and in terms of shape data, why did they perform relatively worse for size than both bone landmarks and the total configuration? Before answering this question, we want to stress again that, although one can try to estimate absolute errors, the most important assessment is always relative. This means that, within the methodological framework that will be used to analyze the data, measurement error should be assessed for the specific measurements under study and in relation to sample variance. For instance, in terms of absolute errors, glabella and soft nasion are two of the most reliable landmarks; orbitale is also highly precise, while porion is not. Thus, one might expect measurements between precise landmarks to be less affected by error compared to distances involving one or more unreliable landmarks. However, if we measured inter-landmark distances between glabella and the other three aforementioned points, ICC would tells us a different story with glabella to nasion (ICC = 0.43) and glabella to orbitale (ICC = 0.72) faring much worse than glabella to porion (ICC = 0.96). Why has this happened? Simply because, compared to longer measurements such as glabella to porion, and regardless of absolute errors, short distances (and thus especially glabella to nasion) are typically much more strongly affected by measurement error in relative terms. This effect, for which a small error is proportionally more relevant as the distance being measured becomes smaller, has been known for a long time, as nicely reviewed by Polly et al.[23].

If we go back to the question of why nasal size is less reliable, it should be clear that this is mainly because the nose is small compared to the rest of the face, thus making errors in the estimate of its size relatively more important. In contrast, the size of the landmark configuration is largely irrelevant for shape data, because size is standardized and therefore Procrustes shape data are scale free. However, as their errors are summed over the entire landmark configuration, especially if sample variance is not particularly large, even small absolute errors can become large, as happened in our study when we used all landmarks or just those on bones.

It should also be borne in mind that our main estimates of the magnitude of measurement error are relative to total sample variance. Taking this into account, we might be able to suggest another reason why reproducibility was higher in nasal shape. This is simply because the nose soft-tissue may vary more among individuals compared to bones. Unfortunately, we cannot assess if this is the case directly, as one cannot compare variance of different landmark configurations[3]. However, we may get a hint to whether this explanation is reasonable by comparing the range of variation within each configuration in relative terms. One approach for doing this is to compute the ratio between the largest and smallest shape distances to the sample mean. This is like expressing the range of a measurement in relative instead of absolute terms. If this is done in our samples (replica or study sample), the largest distance for nasal shape can be up to eight times larger than the smaller, whereas for the total and bone landmark configurations the range is only two to four times. This difference in the relative range of variation is robust to the presence of outliers (which may strongly influence estimates of ranges), as using trimmed ranges that leaves out the 5% most extreme individuals within each set of data, the relative range of shape distances for the nose is approximately four but it is just about two for the two other configurations. Thus, regardless of the effect of extreme observations, the nose seems to vary twice as much as the other landmarks.

### Assessing measurement error in PGMM studies of MRIs

Despite more than two decades of applications of PGMM to MRI data, and a large number of studies, most of the PGMM analyses in the literature did not explicitly mention whether measurement error was assessed, and when it was done, often only absolute errors were reported[17, 18]. For instance, Bookstein et al.[4] stated that average inter-operator differences in digitized landmarks (averaging over 5 randomly selected subjects) ranged from 0.5 mm to 2.0 mm for most landmarks, and thus they decided to have all landmarks digitized by a less experienced operator reviewed by a more experienced one. By doing so, they presumably removed the bias and obtained reliable landmarks for comparing groups. The magnitude of the absolute differences they found is similar to the one we observed on average. This seems to suggest that the amount of inter-operator bias we found is not atypical. However, absolute errors, although informative, fail to relate the magnitude of measurement errors to that of the ‘real’ biological variation being studied. Indeed, although they did not report how relative error was assessed, Bookstein et al.[4] concluded that, for semilandmarks in their study, error was “comparable to the standard error of the better landmark points and … considerably smaller than the magnitude of the effects reported in the present analyses (reported in units of Procrustes distance, not mm)”.

Gharaibeh et al.[36] also used MRIs to compare normal and diseased groups mostly to explore whether time from the onset of schizophrenia produced significant changes in brain morphology. They confirmed some specific localized differences related to schizophrenia, which had been detected by the pioneering work of DeQuardo et al.[37], and suggested that their work was consistent with previous research showing that brain plasticity in schizophrenic patients is different than in normal individuals. However, the detailed protocol of Gharaibeh et al.[36], which included randomization of MRIs to keep operators blind to information on groups and to control for time effects in the digitization, did not include an assessment of measurement error. Presumably, they assumed that, using a single operator, errors would be small relative to sample variance and group differences, and the same assumption probably was made in other PGMM MRI studies [38] that did not report measurement error. Although this assumption may sound reasonable, one should test it at least in an appropriate subsample and insure that the observed measurement error is representative of the main study.

Sometimes landmarks are placed on images by an algorithm instead of a human operator. This may increase precision but could introduce large inaccuracies. For instance, Marečková et al.[39], in a study on sex differences in human faces, used an automatic procedure to place landmarks on 3D images reconstructed from MRIs. They computed absolute differences between landmarks placed by the automatic procedure with those digitized manually on the same image by an operator and reported differences ranging from about 1.6 mm to 1.1 cm. They argued that the systematic error by the automatic procedure was more desirable than low precision landmarks placed by a human operator. However, they did not relate either source of error to sample variance and the effect size of the factor they were measuring, which inevitably prevents the assessment of the effect of misplacing landmarks up to more than 1 cm from where a human operator would have digitized them.

In contrast to most of the aforementioned studies, Weinberg et al.[40] and Barbeito-Andrés et al.[7] provided estimates of reproducibility in a way which relate them to sample variance. First, they reported ICC for landmark coordinates from repetitions of the digitizations. Weinberg et al.[40] computed ICC directly from raw landmark coordinates. In contrast, Barbeito-Andrés et al.[7] did it using Procrustes shape coordinates. This second option seems more reasonable, as, using raw coordinates, size and shape components of form have not been separated and sample variance is inflated by (generally) biologically meaningless nuisance parameters (i.e., translational and rotational differences).

In fact, despite being done after removing size and positional differences, even using Procrustes shape coordinates for computing univariate ICCs for each of them, as in Barbeito-Andrés et al.[7], is not the most desirable option. This is for two reasons: 1) the data are multivariate and the error should be assessed in terms of how it simultaneously affects all variables; 2) the Procrustes superimposition is a convenient but arbitrary choice, which precludes any analysis of variation at specific landmarks [11, 41]. This second issue requires a brief clarification of what we mean by “arbitrary choice”. Procrustes is simply a least square method to minimize positional differences after mean centering the data and standardizing size. It is not a model of biological variation but it has been shown that, as long as all analyses and interpretations are considering simultaneously all landmarks, results are likely accurate[1]. To appreciate why reporting ICC one coordinate at a time is undesirable one can use an intuitive 2D example (as in Fig 9 of Viscosi & Cardini [11]. With 2D data, the Bookstein’s baseline superimposition[42] offers an alternative method for registering the data, which is simpler, more intuitive and, for a reasonable choice of the baseline, as accurate as the Procrustes methods. Thus, after selecting two points, all individual configurations are rescaled, translated and rotated so that those two points overlap. This removes positional and size differences and creates a new set of shape coordinates. Now, if one decided to report ICC from replicas of data superimposed using Bookstein’s baseline method, the two baseline points would have ICC of exactly one. This is not because they have higher precision but simply because of an artifact due to the arbitrariness of the superimposition. Although with Procrustes, being a least square method, differences are spread over all landmarks in a configuration, so that none will have ICCs of exactly one, as in the baseline example, the problem is the same: those univariate ICCs are, to a smaller or larger extent, a by-product of the choice of the superimposition and are therefore unreliable.

However, Barbeito-Andrés et al.[7] also assessed measurement error in a correct and fully multivariate fashion using the same ANOVA model as we did. These results were reported in their paper and allowed to compute both R2 for the sum of squares and the multivariate ICC for the symmetric component. This shows that both R2 and ICC are high for the symmetric component of individual shape (respectively, 84% and 0.93). Measurement error accounts for just 7% of variation. However, despite finding significance for fluctuating asymmetry, the magnitude of fluctuating asymmetry is the same (7%) as measurement error, which suggests that analyses at individual level will be fine whereas the interpretation of asymmetric patterns may require great caution, as we stressed in the preliminary considerations. Indeed, that P values may be less important than the size of the effect they are testing was also implicit in our own analysis, which showed significantly larger individual variation in all ANOVAs despite inter-operator differences accounting for up to 32% of sample variance.

## Conclusions and recommendations

Our study shows that measurement error is crucial in PGMM studies using MRIs, and as already stressed in a broader context[43], it must be done in a way which is specific to the type of data and the set of methods being employed. For instance, the precision of raw landmark coordinates, or their inter-landmark distances, does not guarantee a negligible error in the Procrustes shape coordinates derived from those same points. Also, absolute error is much less relevant than relative error, in which the importance of measurement error is assessed in relation to the amount of variation in the sample. Finally, and critically, consistently with findings from other recent PGMM studies[14, 15], when data from different operators are used, inter-operator differences must be quantified before one can decide whether it is appropriate or not to merge them in a single dataset. As we showed, in this respect, size and shape may behave differently and, again, as in the two recent studies we cited, different subsets of landmarks may be more or less affected by measurement error. Indeed, by comparing landmarks on hard and soft tissues we did find that errors were larger on bones, as expected in MRI data, but also unexpectedly found that this only applied to shape and not size, a finding that we hope will stimulate future studies of the same type in order to assess whether this is specific to our dataset or a common pattern in MRI PGMM analyses of relatively small structures.

Likely, assessing measurement error, and especially inter-operator (and/or instrumental) biases, will become more important as 'big data' becomes more accessible. Data sharing via online databases or journal webpages will also contribute to make more data available. Sometimes, however, as often in the case of data coming from different sources, one might not have the information to assess error, which leaves open the question of whether the combined dataset might be reliable. Alternatively, in a more optimistic scenario, if data to assess biases are available, one could potentially quantify and try to control those errors, but, in order to do that accurately, very large samples with replicas might be necessary.

In conclusion, to summarize the main points on reproducibility in PGMM studies on MRI data, we recommend the following:

1. Always test measurement error at least in a representative subsample before proceeding with a bigger study. Although it cannot replace an accurate test of error using replicas, exploring any suspicious pattern of group structure by plotting the data using different symbols for different operators (or instruments etc.) may also provide interesting preliminary clues on potential problems with measurement error, as exemplified in Fig 6.
2. Whenever possible, try to include in the assessment all sources of errors (device and operator, intra- and inter-operator etc.) or at least the error source which is presumably larger.
3. Explore absolute errors to flag, and potentially exclude, unreliable landmarks while bearing in mind that some of the conventional methods may be inappropriate for raw landmark coordinates
4. Perform fully multivariate analyses for Procrustes shape data and, using superimposed data, avoid analyses and interpretations of single coordinates or landmarks.
5. Relate measurement error to the magnitude of the effect being tested (as using R2 or ICC—both of them multivariate in the case of shape data) and bear in mind that effect sizes might be more important than P values.
6. If a larger configuration of landmarks is split into subsets (e.g., soft- *vs* hard-tissues; different regions of the head or developmental modules etc.), measurement error should be re-assessed in each subset.
